# Providing Psychological Support to Parents of Childhood Cancer Survivors: ‘*Cascade*’ Intervention Trial Results and Lessons for the Future

**DOI:** 10.3390/cancers13225597

**Published:** 2021-11-09

**Authors:** Claire E. Wakefield, Ursula M. Sansom-Daly, Brittany C. McGill, Kate Hetherington, Sarah J. Ellis, Eden G. Robertson, Mark W. Donoghoe, Maria McCarthy, Lauren Kelada, Afaf Girgis, Madeleine King, Martha Grootenhuis, Antoinette Anazodo, Pandora Patterson, Cherie Lowe, Luciano Dalla-Pozza, Gordon Miles, Richard J. Cohn

**Affiliations:** 1 School of Women’s and Children’s Health, UNSW Medicine and Health, UNSW Sydney, Kensington, NSW 2052, Australia; ursula@unsw.edu.au (U.M.S.-D.); b.mcgill@unsw.edu.au (B.C.M.); k.hetherington@unsw.edu.au (K.H.); sarah.ellis@unsw.edu.au (S.J.E.); eden.robertson@unsw.edu.au (E.G.R.); m.donoghoe@unsw.edu.au (M.W.D.); l.kelada@unsw.edu.au (L.K.); Antoinette.Anazodo@health.nsw.gov.au (A.A.); r.cohn@unsw.edu.au (R.J.C.); 2 Kids Cancer Centre, Sydney Children’s Hospital, Randwick, NSW 2031, Australia; 3 Nelune Comprehensive Cancer Centre, Prince of Wales Hospital, Randwick, NSW 2031, Australia; 4 School of Psychology, University of Sydney, Sydney, NSW 2006, Australia; madeleine.king@sydney.edu.au; 5 Stats Central, Mark Wainwright Analytical Centre, UNSW Sydney, Sydney, NSW 2052, Australia; 6 Clinical Sciences, Brain and Mind, Murdoch Children’s Research Institute, Melbourne, VIC 3052, Australia; Maria.McCarthy@rch.org.au; 7 Department of Paediatrics, University of Melbourne, Melbourne, VIC 2052, Australia; 8 South West Sydney Clinical Campuses, UNSW Medicine and Health, Sydney, NSW 2052, Australia; afaf.girgis@unsw.edu.au; 9 Princess Máxima Center for Pediatric Oncology, 3584 CT Utrecht, The Netherlands; m.a.grootenhuis@prinsesmaximacentrum.nl; 10 Research, Evaluation and Social Policy Unit, Canteen, Newtown, NSW 2042, Australia; pandora.patterson@canteen.org.au; 11 Sydney Nursing School, Faculty of Medicine and Health, University of Sydney, Sydney, NSW 2006, Australia; 12 Queensland Children’s Cancer Centre, Queensland Children’s Hospital, South Brisbane, QLD 4101, Australia; clowe@csys.com.au; 13 Cancer Centre for Children, The Children’s Hospital at Westmead, Westmead, NSW 2145, Australia; luciano.dallapozza@health.nsw.gov.au; 14 Child and Adolescent Mental Health Service, Perth Children’s Hospital, Perth, WA 6009, Australia; gordon.miles@health.wa.gov.au

**Keywords:** childhood cancer, parent, feasibility, acceptability, efficacy, psychological interventions, videoconferencing, survivorship, quality of life, cognitive behavior therapy

## Abstract

**Simple Summary:**

We assessed a new group-based cognitive behavior therapy videoconferencing program to support parents of childhood cancer survivors. The trial allocated parents to three groups: Cascade, peer-support, waitlist. Cascade achieved good parent engagement. We successfully delivered Cascade to participants who lived >3200 km apart. Any technical difficulties caused only minor disruptions. Most Cascade parents were satisfied and reported experiencing benefits from the program. However, Cascade did not improve our main outcomes, including parents’ quality of life, depression and anxiety. Cascade parents reported a short-term improvement in their confidence to use the skills they learnt, but this did not translate into actual use. After six months, Cascade parents felt their child survivor had lower psychological health than waitlisted parents. Our findings show that while some parents find Cascade helpful, it may not suit everyone. We used these findings to further improve Cascade and will trial the new version in future.

**Abstract:**

We conducted a three-armed trial to assess Cascade, a four-module group videoconferencing cognitive behavior therapy (CBT) intervention for parents of childhood cancer survivors currently aged <18 years. We allocated parents to Cascade, an attention control (peer-support group), or a waitlist. The primary outcome was parents’ health-related quality of life (*PedsQL-Family Impact/EQ-5D-5L*) six months post-intervention. Parents also reported their anxiety/depression, parenting self-agency, fear of recurrence, health service and psychotropic medication use, engagement in productive activities, confidence to use, and actual use of, CBT skills, and their child’s quality of life. Seventy-six parents opted in; 56 commenced the trial. Cascade achieved good parent engagement and most Cascade parents were satisfied and reported benefits. Some parents expressed concerns about the time burden and the group format. Most outcomes did not differ across trial arms. Cascade parents felt more confident to use more CBT skills than peer-support and waitlisted parents, but this did not lead to more use of CBT. Cascade parents reported lower psychosocial health scores for their child than waitlisted parents. Cascade parents’ health service use, psychotropic medication use, and days engaged in productive activities did not improve, despite some improvements in waitlisted parents. Our trial was difficult to implement, but participants were largely satisfied. Cascade did not improve most outcomes, possibly because many parents were functioning well pre-enrolment. We used these findings to improve Cascade and will trial the new version in future.

## 1. Background

Significant improvements in childhood cancer treatments have resulted in growing numbers of young survivors worldwide [1,2]. Despite the benefits of cancer treatment, this can be a vulnerable time for parents/caregivers, who can experience poor quality of life (QOL),_ENREF_9 anxiety, depression, loneliness _ENREF_8 [3,4,5,6,7], and fear of their child’s cancer recurring [4,5]. Without intervention, parents’ distress can last years [5,8]. Families in rural or remote settings have less access to psychosocial support [9,10] and experience more cancer-related financial impacts [11] than metropolitan families, placing them at greatest risk of poor outcomes [12,13]. Some evidence suggests that parents’ (particularly mothers’) QOL is closely associated with their child’s QOL [5,14,15,16]. Poor parent mental health may compromise parenting skills and may subsequently exacerbate anxiety, depression,_ENREF_24 and behavioral problems in survivors and siblings [15,17,18,19,20].

Given parents’ risk of psychological difficulties, international standards of care state that parents should have early and ongoing access to psychosocial support and evidence-based interventions throughout their child’s cancer trajectory [21]. Psychological interventions can be effective for parents of children with cancer and other serious illnesses [22,23], yielding small–moderate improvements in QOL, anxiety, and other outcomes [23,24,25,26,27,28,29]. Cognitive behavior therapy (CBT) interventions that increase parents’ use of adaptive coping strategies may also enhance their parenting skills, reduce parents’ risk of depression/anxiety, and in turn benefit the whole family [15,19,30]. However, few interventions have implemented evidence-based treatments [31,32] and many have not been evaluated with rigorous research designs in pediatric oncology [7,24,25,27]. With the exception of the eSCCIP [23], most interventions are also implemented face-to-face, limiting benefits to rural-remote families [7,25,27,33].

To fill these gaps, our team developed ‘*Cascade’* [‘Cope, Adapt, Survive: life after cancer’], a group-based videoconferencing intervention grounded in CBT principles. The Cascade pilot indicated that the program was feasible and acceptable to parents whose children were treated at one metropolitan hospital [34]. The Cascade trial expanded upon the pilot to answer these questions:

1. Is it feasible to conduct a national trial of Cascade, indexed by timeliness and trial participation?
2. Is it feasible to simultaneously deliver Cascade to parents of children treated at multiple cancer centers across Australia (eight hospitals across five states), indexed by treatment fidelity, participant engagement, reach, organizational impacts and technical difficulties?
3. Is Cascade acceptable to parents of children treated at multiple cancer centers across Australia, assessed through intervention satisfaction, perceived benefit/burden, group cohesion between group members and working alliance with the psychologist?
4. Is it psychologically safe to deliver Cascade to parents of children treated at multiple cancer centers across Australia from one lead site (assessed by between-session distress ratings)?
5. Compared with an attention control (videoconferencing peer-support group) and a waitlist control, is Cascade able to:
  - Improve parents’ health-related QOL (HRQL, *primary outcome*)?
  - Improve parents’ psychological outcomes (depression, anxiety, fear of cancer recurrence, perceived parenting self-agency)?
  - Increase parents’ confidence to use CBT skills and actual use of CBT skills?
  - Improve parent-reported HRQL in their child who had cancer?

We also assessed the impact of Cascade on parents’ use of mental health services, other health services, and medications, and their general functioning (i.e., engagement in productive activities such as paid work, hobbies, and socializing), relative to the waitlist and peer-support group conditions. 

## 2. Materials and Methods

### 2.1. Study Design

This single-blind phase-II randomized trial used three arms to compare Cascade with a videoconferencing peer-support group and six-month waitlist control (1:1:1 allocation). This study was conducted according to the guidelines of the Declaration of Helsinki and approved by the Institutional Review Board of the Hunter New England Ethics Committee (protocol code: HREC/14/HNE/44, approved 13 March 2014). The trial was registered on the Australian/New Zealand Clinical Trials Registry (ACTRN12613000270718). With ethical approval, some aspects of the study design were modified after publication of the study protocol [35] as challenges were identified in the roll-out of the trial. All study design challenges, resulting protocol modifications, and the impact of the modifications made are listed in Supplementary Table S1. This paper reports on the final, amended study design.

### 2.2. Participants

Informed consent was obtained from all participants involved in this study. Parents/caregivers were eligible if they (i) had a child aged < 18 years who had completed cancer treatment with curative intent in the last 10 years, (ii) were able to communicate in English, and (iii) could give informed consent. Parents were ineligible if their clinical team reported (or parents reported to the research team) that they (i) were currently experiencing severe depression/suicidality or substance abuse or (ii) had experienced psychosis.

### 2.3. Procedures

We recruited parents through eight Australian hospitals from September 2014 to January 2018 (before the COVID-19 pandemic began). Site investigators (oncologists/social workers) identified parents via electronic medical records. We mailed an invitation letter, consent form, and reply-paid envelope to eligible parents. We made two follow-up calls and sent one text message reminder to parents who did not respond, before considering them non-respondents. We also advertised via newsletters/social media of relevant cancer organizations. The study research officer telephoned each parent who opted in to check their access to required equipment and provide education about using the online platform (WebEx^TM^). A study psychologist then completed an introductory phone call to each participant, to introduce them to this study and answer any questions. All Cascade and peer-support sessions were delivered virtually from the Kids Cancer Centre at Sydney Children’s Hospital in NSW, Australia.

### 2.4. Study Arms

#### 2.4.1. Cascade

Cascade is a manualized program comprising four 90 min videoconferencing modules and a one-to-one follow-up “booster” session (content is summarized in Table 1). Each module focuses on common challenges for parents, derived from in-depth qualitative studies [36,37,38,39] and a systematic review [4]. Cascade was designed to serve as a ‘selective preventative’ [40] program, as it was specifically targeted toward parents at a time that is recognized to be vulnerable to mental health challenges, i.e., after their child’s cancer treatment (‘selective’), but was open to parents who were not currently clinically distressed (‘preventative’) [4,41]. We did, however, exclude parents experiencing severe psychopathology because it was deemed that their needs would be best addressed through more intensive, one-to-one, services. 

#### 2.4.2. Attention Control (Peer-Support Group)

We delivered peer-support groups in an identical manner to Cascade groups using the same online platform, same delivery schedule, and same psychologists. However, the psychologists provided supportive, non-directive peer discussion on the same topics covered in Cascade (e.g., ‘relationships after cancer’) [42]. Psychologists did not teach CBT skills in this condition. We used an attention control to control for parents’ expectations of receiving some form of treatment and to hold constant the treatment contacts and peer-peer interaction received by participants. We offered optional group booster sessions for peer-support group participants four weeks after program completion.

#### 2.4.3. Waitlist Controls

Participants who were allocated to the waitlist control received standard care for six months, before being randomized to Cascade or a peer-support group. 

### 2.5. Randomization of Groups and Blinding

An independent researcher at Sydney Children’s Hospital used an electronic randomizer to generate two separate algorithms. The first algorithm generated a random sequence of the three trial arms (Cascade, peer-support, waitlist). Groups of five consecutively recruited participants were allocated to trial arms according to this random sequence. This group-based randomization was suited to the group-based nature of the Cascade and peer-support group trial arms and minimized the time delay for participants to begin participating in the trial. The second algorithm was generated for participants initially allocated to the waitlist condition, to allocate them to either Cascade or peer-support group at the end of their waitlist period. Re-randomized (waitlist) participants could be allocated to join groups with participants who were being randomized for the first time (this occurred five times over the trial; their data were not used to assess the efficacy of the Cascade or peer-support group interventions).

Participants in the active treatment arms were blinded to their group allocation (i.e., parents did not know whether they were allocated to Cascade or to the peer-support group); however, the psychologists and research officers could not be blinded due to the distinctiveness of the Cascade and peer-support group training manuals/resources. Three psychologists delivered both interventions (Cascade and peer-support group) to reduce confounding of any psychologist-specific delivery factors. Groups were allocated to psychologists based on their workload/availability.

### 2.6. Measures

We collected participant data at: baseline (Q1), 2–4 weeks post-intervention (Q2), 2–4 weeks post-booster (Q3) and 6 months post-intervention (Q4) (see Table 2 for details). Waitlisted participants completed questionnaires at the same time points as Cascade/peer-support group participants, plus one additional questionnaire after completion of their active treatment period (Q5).

#### 2.6.1. Feasibility of Conducting the National Trial

We collected the following data on the feasibility of conducting the national trial: timeliness (indexed by time taken to recruit sufficient participants and time taken to complete Q1 and commence a group-based intervention), trial participation (indexed by trial response rate, for which the target was >40% [35]), trial enrolment rate (percent of parents who progressed to randomization after opting in, with no target set before the trial), and the trial attrition rate (the target was <20% [35]). We could not assess the trial response rate at every site because some hospital ethics review boards had policies that did not provide approval to collect data about non-respondents. We also included open-ended advertisements for this study on social media which further reduced our ability to calculate an accurate response rate. We were, however, able to calculate the response rate for families from Sydney Children’s Hospital.

#### 2.6.2. Feasibility of Delivering Cascade Nationally

We assessed the feasibility of delivering Cascade, including assessing: fidelity of the treatment when delivered by multiple psychologists (independent raters viewed a random selection of 30% of all sessions, nominated which group the sessions were part of and rated the extent to which the session content adhered to the Cascade manual) and participant engagement across sites (total sessions attended/parent engagement in home practice activities).

We also assessed intervention reach (indexed by the proportion of parents participating from regional/remote regions and the number of inter-state sessions delivered) and organizational impacts (the proportion of sessions occurring out of business hours). We also assessed technological difficulties (time to commence each session, proportion of participants requiring loaned equipment, any technological issues and their perceived disruptiveness).

#### 2.6.3. Acceptability/Satisfaction

We assessed parents’ satisfaction with key elements of Cascade using 10 adapted intervention satisfaction items [44]. These items provided a new depth of data that was not collected in the pilot [34]. We also collected parents’ ratings of whether Cascade/peer-support group was beneficial or burdensome in any way (0 = ‘not at all’ to 4 = ‘very much’), with an open-ended question inviting further elaboration. We also assessed parents’ perceived group cohesion between group members [46] and their perceived working alliance with the psychologist [47].

#### 2.6.4. Safety

We assessed the psychological safety of Cascade by administering the Emotion Thermometers [48] each week, between every module (categorizing scores of ≥7/10 and an increase of more than three points from the previous score as clinically concerning events).

#### 2.6.5. Efficacy

The primary outcome was parents’ health-related quality of life (HRQL), assessed using the *PedsQL Family Impact Module* [49], and the *EQ-5D-5L* [50]. The *PedsQL Family Impact Module* was specifically designed to measure the impact of childhood chronic health conditions on parents and the family unit. The core domains include parent-reported physical, emotional, social, and cognitive functioning, which can be summed to create a parent HRQL summary score. The *EQ-5D-5L* is a generic preference-based instrument which includes five dimensions (mobility, self-care, usual activities, pain/discomfort and anxiety/depression), each measured at five levels (no problems, slight problems, moderate problems, severe problems and extreme problems). 

Secondary outcomes included parents’ psychological outcomes (communication, worry about child’s health, daily activities and family relationships [49], anxiety and depression [52], parenting self-agency [53], and fear of cancer recurrence [54]). We assessed parents’ confidence to use, and actual use of, CBT skills, adapted from a previous study [57]. We also assessed parent-reported HRQL of their child who had cancer using the PedsQL parent-proxy[55] which produces two HRQL summary scores: physical functioning and psychosocial functioning (comprising three dimensions: emotional, social and school functioning).

All HRQL and psychological outcomes assessed with validated measures were scored according to standard scoring algorithms provided by the instrument developers. *EQ-5D-5L* scores were calculated using an Australian value set [58].

We assessed parents’ use of mental health services (psychologist, social worker, counselor, psychiatrist or cancer support organization) and other health services (general practitioner, oncologist/radiation oncologist, nurse in hospital, or nurse in the community), and the number of times parents had visited a hospital emergency department or been admitted to hospital in the last six months. Parents also listed their regular psychotropic medications and supplements in the last four weeks, including reasons for use. Finally, we measured parents’ general functioning (indexed by the number of days they spent engaging in productive activities in the last four weeks).

#### 2.6.6. Measures Not Reported in This Paper

Before randomization, a research officer administered the Psychosocial Adjustment to Illness Scale (PAIS) Interview [59] over the telephone. The PAIS assesses adjustment of carers to illness across seven domains. Data from the PAIS were used to develop an understanding of parents’ baseline levels of adjustment (at Q1) and have been reported elsewhere [60,61,62]. The PAIS was administered once, before randomization, so it was not used to assess the efficacy of Cascade.

### 2.7. Data Analysis

We conducted analyses using SPSS (v24.0) [63] and R (v3.6.1) [64]. We used descriptive statistics such as medians and percentages to examine the sample’s demographic composition and to evaluate feasibility, acceptability and safety. We used a Wilcoxon signed-rank test with continuity correction to assess working alliance changes over time within each group. The primary efficacy endpoint was HRQL 6 months post-intervention (Q4), assessed by *PedsQL* and *EQ-5D-5L*. Based on intention-to-treat principles, we used linear mixed models with participant-specific random intercepts to estimate the trajectories of parents’ scores on the HRQL scales, Emotion Thermometers, depression, anxiety and parenting self-agency. We used a mixed effects proportional odds model to analyze fear of recurrence, and mixed-effects logistic regression models for CBT skills use, and mental health and other health service use. We analyzed medication use as binary (any medications) and count (number of medications) outcomes, using mixed-effects logistic and Poisson regression models, respectively. We used Kruskal–Wallis tests to examine differences between arms in changes from baseline (Q1) in parents’ general functioning. We transformed PROMIS Anxiety and Depression scores into t-scores.

To assess treatment fidelity, five independent raters randomly selected 14 sessions to rate (30% of sessions; 14/47 sessions, two raters per session). Raters had completed at least an undergraduate degree in psychology.

For mental health and other health service use in the last six months, we only analyzed data from Q1 and Q4 to avoid overlaps in parents’ recall periods (e.g., at Q2, recall of service use in the last six months would overlap with that reported in Q1). Given the small cell sizes at the individual professional level, we grouped professions into two broad categories (mental health professionals; other health professionals) for analyses. A medical student categorized medications by therapeutic class and purpose, in line with the MIMS Online database, with supervision from a senior pediatric oncologist (RJC).

Baseline between-group differences at Q1 were implicitly adjusted for in the longitudinal models (see Table 3 for baseline data presented by group). We did not adjust analyses to account for the fact that two parents of the same child could participate because this occurred only three times (i.e., three couples participated), making it difficult to estimate and account for possible correlation given such a small number of clustered observations. Given the small sample and the brief responses provided by parents, we conducted a directed qualitative content analysis [65] of open-ended question responses. 

Our target sample size was 120, which would have provided 80% power to detect medium-large differences in HRQL (an effect size of 0.65) [35]; however, recruitment was closed when the funding period ended. We used a significance level of 0.05 throughout this paper and did not adjust for multiple comparisons.

**Figure 1:**
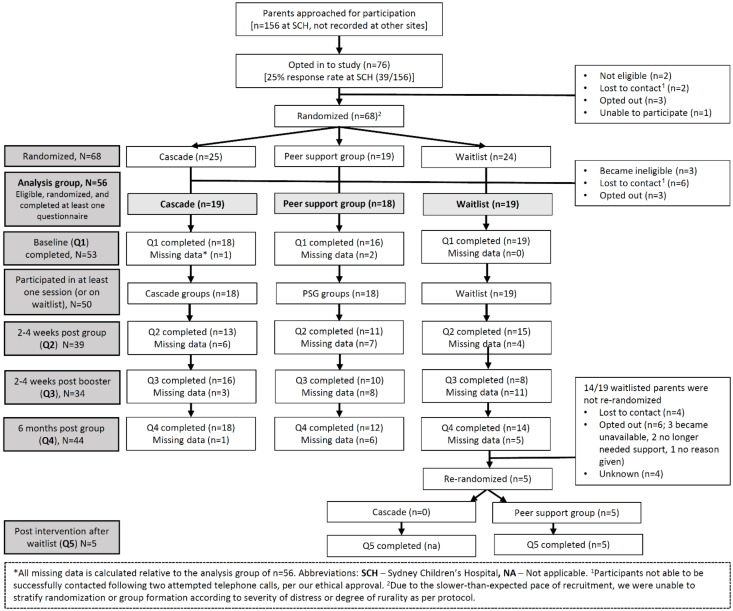
Rates of recruitment and retention over the course of the *Cascade* study.

Figure 1 summarizes participants’ flow-through and retention across the trial.

### 2.8. Feasibility of the National Trial

Table 4 summarizes the feasibility and acceptability data collected in the trial. Recruitment for the trial continued over 39 months (our original target was 15 months). The median time from completion of Q1 to group commencement was 35 days (SD 57).

At Sydney Children’s Hospital, 39 parents opted in, out of 156 parents who were invited (trial response rate at primary site: 25%; original target >40%). Across all recruiting sites, 76 parents opted into this study, 68 of whom were randomized (trial enrolment rate: 90%). The trial was opened first at Sydney Children’s Hospital, with other sites joining the trial once ethical and site-specific approvals were obtained.

Of the 68 randomized participants, 56 commenced this study, 53 completed Q1 and 39 participants completed Q2 (original target: 120 participants). Thirty-four participants completed Q3 and 44 participants completed Q4. The study attrition rate was therefore 21% (total Q1 completions-total Q4 completions/total Q1 completions: 56 − 44/56 * 100; original target <20%). Attrition appeared somewhat different across groups, at 5% for Cascade, 33% for the peer-support groups and 26% for waitlisted parents. 

### 2.9. Feasibility of Delivering Cascade Nationally

Participants: Fifty-three parents provided their demographic details in Q1 (Table 3 reports data on all 56 parents who were allocated to groups, in accordance with intention-to-treat principles). Most participants were mothers (49/56, 88%), with a median age of 40 years (interquartile range [IQR] = 38–45). The median time since the child’s cancer treatment completion was 14 months (IQR = 9–21). Twenty-five parents were allocated to Cascade, 18 of whom completed Q1 and attended at least one Cascade session (72%). Nineteen parents were allocated to a peer-support group, 16 of whom completed Q1 and 18 of whom attended at least one peer-support group session (95%). Twenty-four parents were allocated to the waitlist, 19 of whom completed Q1. 

We ran six Cascade groups (delivered via 23 group sessions plus one set of individual sessions when a group session was cancelled) and six peer-support groups (delivered via 24 sessions). Following the 6 month wait, 5/19 waitlisted parents (26%) wished to complete the intervention, with all parents randomly allocated to the peer-support group and beginning the peer-support group (100%). (Data from the PSG for these five parents are included in the acceptability and psychological safety data below, but not in the efficacy data as these parents only completed one final questionnaire, Q5.)

Treatment fidelity: Adherence to the Cascade manual was high, with key Cascade-specific intervention components covered well in Cascade sessions and not in the peer-support group sessions. Raters correctly identified which arm all sessions were from (100%; 14/14). Ratings indicated that Cascade-specific intervention components were covered well in Cascade sessions (the raters indicated that 85% of intervention components were addressed to an acceptable degree; mean score of 4.7/7; SD:1.83). Cascade-specific components were not covered in almost all peer-support group sessions (raters indicated that 94% of the Cascade intervention components were not acceptably covered in the peer-support group sessions; mean score of 0.6/7; SD:1.32).

Participant engagement: Participant session engagement was high for both intervention arms, with most parents attending all sessions (see Supplementary Figure S1). The median session length for Cascade was 91 min (range: 60–115; target: 90). The median session length for the peer-support group was 88 min (range: 60–104, target: 90). Nine Cascade parents reported completing at least some of the homework set after module 1 (9/14 parents who completed the homework questionnaire, 64%); 10 parents reported completing at least some of the module 2 homework (10/14, 71%); 9 parents reported completing at least some of the module 3 homework (9/14, 64%); and 10 parents reported completing at least some of the module 4 homework (10/14, 71%).

Reach: Participants resided a median 29 km (18 mi) from their state capital city (IQR = 12–103). Just over one quarter of Cascade participants resided in regional/rural areas across Australia (5/19; 26%). We successfully delivered Cascade groups to participants who lived >3200 km apart (2011 mi), with two groups including participants from three states together. 

Organizational impacts: Almost half of all sessions were conducted out of business hours (49%), representing approximately 36 h of the psychologists’ time.

Technological difficulties: The median time for each session to commence was 3 min (range = 0–50). Seventy-four percent of sessions had all participants log-in within 5 min of the scheduled start time. While technological difficulties were common (66% of all sessions), most disruptions were minor (median disruptiveness score: 2/10; range: 1–7). 

### 2.10. Acceptability

**Figure 2:**
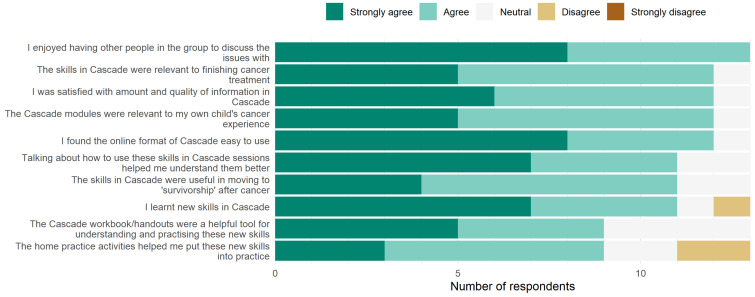
Parents’ satisfaction ratings for various aspects of Cascade.

Intervention satisfaction (Figure 2): Overall, satisfaction ratings were high. Among respondents at Q2, all Cascade parents agreed or strongly agreed that they ‘enjoyed having other people to discuss issues with’ (13/13; 100%). Almost all parents indicated that the ‘online format was easy to use’, the ‘modules were relevant to their child’s cancer experience’, they were ‘satisfied with the amount and quality of information provided’ and ‘the skills learnt were relevant to their child finishing cancer treatment’ (12/13; 92%). Ratings for the home activities and workbook/handouts were somewhat less positive (9/13; 69% of parents agreed that ‘the home activities helped put skills into practice’ and that ‘the workbook/handouts were helpful tools’).

Perceived benefit/burden: A total of 13 Cascade parents and 15 parents completed the Q2 benefit/burden items. Of these, most parents reported that Cascade was ‘quite a bit’ to ‘very’ beneficial and few reported that participation was burdensome. Supplementary Table S2 summarizes parents’ responses to the open-ended questions. Parents described Cascade as “rewarding”, “supportive” and “meaningful”. Benefits included connecting with others in a similar situation, normalizing experiences, and learning new skills. Parents noted the convenience of being able to participate from home. Time was the most frequently reported burden. Three parents noted that the sessions reminded them of their child’s difficult cancer experiences, noting that talking about their experiences ‘opened up all the feelings again’ and that it reopened a ‘can of worms’. These parents indicated that discussing their own experiences in an open forum, or hearing others’ stories, was ‘very stressful’ and caused anxiety, and in one case, the parent felt unprepared for this experience. Three parents noted that they participated to support research, rather than expecting therapeutic benefit themselves.

Group cohesion: Cascade group cohesion ratings were high [median score: 6.2/7, range = 4.5–6.8], as were ratings for the peer-support group [median score: 6.0/7, range = 5.1–6.4] (Supplementary Figure S2).

Working alliance: Cascade working alliance ratings were positive, with 87% of parents feeling confident in their group leader’s ability to help them, and 93% reporting that their group leader appreciated them (Supplementary Figure S3). Peer-support group working alliance ratings were also positive.

### 2.11. Psychological Safety

Between-session Emotion Thermometers data indicated that seven Cascade parents (7/19, 37%) and six peer-support group parents (6/23 who participated in the peer-support group either immediately [*n* = 18] or post-waitlist [*n* = 5], 26%) reported a clinically concerning event on at least one occasion over the intervention period. Upon the psychologist follow-up call, no parents were at immediate mental health risk. There was no evidence that average scores for each Emotion Thermometers domain diverged from a linear (straight) line trend over time for either arm (Supplementary Figure S4).

### 2.12. Efficacy

#### 2.12.1. Primary Outcome: Parents’ Health-Related Quality of Life (HRQL)

**Figure 3:**
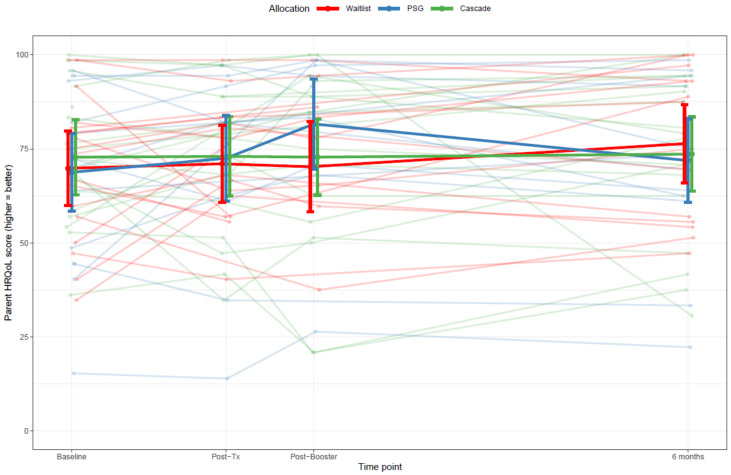
The PedsQL Family Impact Module health-related quality of life scores for parents in Cascade, peer-support and waitlisted groups across each time-point. Notes: The PedsQL HRQL Summary Score is computed as the sum of the items divided by the number of items answered in the physical, emotional, social and cognitive functioning subscales (for this study, only 18 of the 20 items were included two items from the physical scale (“I get headaches” and “I feel physically weak”) were not asked). If more than 50% of the items in the scale were missing, the summary score for that participant not computed at that time point. The above graph shows the individual scores over time, as well as the average scores (with 95% CI) in each study arm in **bold**. A 5-point response scale was utilized (0 = never a problem to 4 = always a problem), and items are reverse-scored and linearly transformed to a 0–100 scale, where higher scores indicate better functioning (i.e., less negative impact). Post-Tx: 2–4 weeks after intervention (Q2).

At baseline (Q1), 80% of parents across all groups had a PedsQL Family Impact Module HRQL Summary Score above 50, indicating a relatively good HRQL (41/51 [missing data for 5 parents]). There was no evidence that parents in Cascade, the PSG, or the waitlist differed in their responses over time for their HRQL as indexed by their PedsQL Family Impact HRQL summary scores (χ^2^(6) = 6.7, *p* = 0.35) (Figure 3); their EQ-5D-5L index scores (χ^2^(6) = 8.0, *p* = 0.24); or their self-reported health via the EQ-VAS (χ^2^(6) = 3.7, *p* = 0.72). 

#### 2.12.2. Secondary Outcomes

##### Parents’ Psychological Outcomes

At baseline (Q1), 31% of parents across all groups had a PROMIS anxiety score of 50 or lower, indicating better (lower) anxiety scores than the average in normative data (16/51). There was no evidence that parents in the three groups had different scores over time for anxiety (χ^2^(6) = 6.5, *p* = 0.37).

At baseline (Q1), 39% of parents across all groups had a PROMIS depression score below 50, indicating better (lower) depression scores than the average in normative data (20/51). There was no evidence that parents in the three groups had different scores over time for depression (χ^2^(6) = 7.2, *p* = 0.30).

There was no evidence that parents in the three groups had different scores over time for communication (χ^2^(6) = 7.9, *p* = 0.24), worry about their child’s health (χ^2^(6) = 10.4, *p* = 0.11), daily activities (χ^2^(6) = 3.4, *p* = 0.76), family relationships (χ^2^(6) = 12.0, *p* = 0.06), parenting self-agency (χ^2^(6) = 11.2, *p* = 0.08), or fear of cancer recurrence (χ^2^(6) = 6.6, *p* = 0.36). 

##### Parent-Reported Child Survivor HRQL

**Figure 4:**
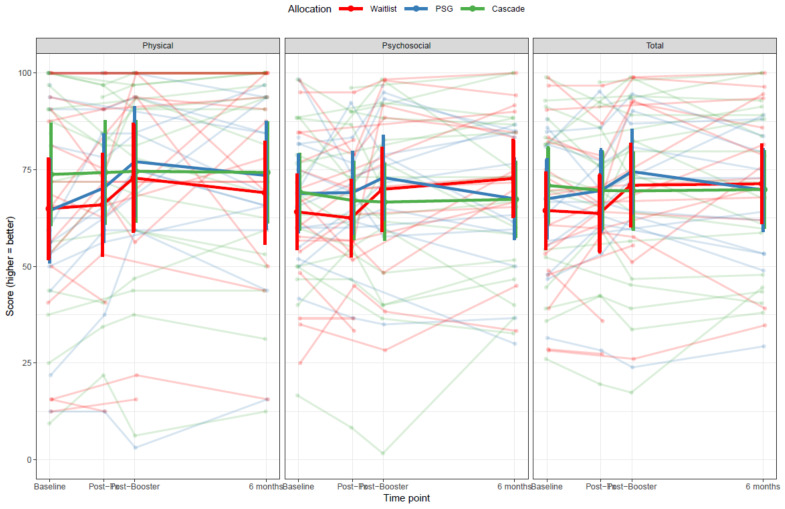
The PedsQL parent-proxy report of health-related quality of life scores for their child who had completed cancer treatment, compared across Cascade, peer-support and waitlisted groups across each time-point The PedsQL parent proxy report contains 21–23 questions about the child’s quality of life across four domains: physical functioning (8 questions), emotional functioning (5 questions), social functioning (5 questions) and school functioning (3–5 questions). The questions are dependent on the child’s age, in categories of 2–4 years, 5–7 years, 8–12 years and 13–18 years. The major difference is that the (pre)school functioning domain contains only three questions for the 2–4 year age group, and five for the older groups. The other differences between age groups are minor: for example, the 2–4 year age group contains a question about picking up toys where the older groups have a question about chores, and the 13–18 year age group asks about “getting along with other teenagers” whereas the corresponding question in the younger groups is about “getting along with other children”. The scoring system is the same across all age groups: original responses are given on a scale from 0 (never a problem) to 4 (almost always a problem) and are reverse-scored and linearly transformed such that 0 = 100 and 4 = 0, and combined to form subscale and summary scores by taking the average score (as long as at least 50 percent of questions are answered). Three summary scores are produced: physical health (including items from the physical functioning subscale), psychosocial health (emotional, social and school functioning subscales) and total (all items). These scores range between 0 and 100, with higher scores indicating better quality of life. Note that two responses in the school functioning subscale were missing and that on five occasions, parents appear to have answered the HRQL questions for 2–4 year olds, even though their children were aged over 4. The above graph shows the individual scores over time, as well as the average scores (with 95% CI) in each study arm in bold.

There was insufficient evidence that parents perceived that their child had a different overall HRQL (summary score) or in the HRQL physical health domain (χ^2^(6) = 12.5; *p* = 0.051; χ^2^(6) = 7.7; *p* = 0.26, respectively) across the three groups between Q1 and Q4. Parents in the Cascade group did, however, report that their child had lower HRQL in the psychosocial health domain compared to the waitlist at 6 months (χ^2^(6) = 14.5; *p* = 0.024). See Figure 4.

#### 2.12.3. CBT Skills Use

**Figure 5:**
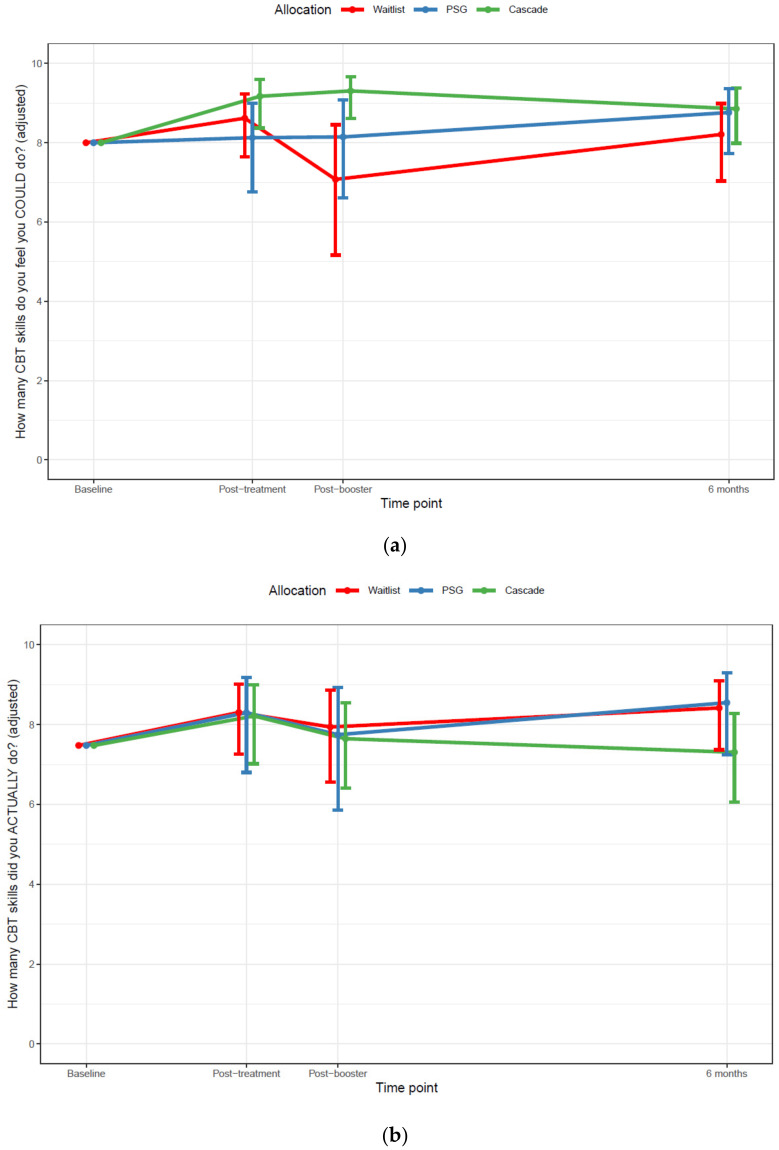
(**a**) Number of cognitive behavioral therapy skills participants felt confident to use, across groups and time points, adjusted for baseline (Q1) differences between groups (**b**) Number of cognitive behavioral therapy skills participants reported that they used within the past four weeks. Notes: The questionnaires at each time point included 10 purposely designed items that assessed participants’ level of confidence in using 10 cognitive and/or behavioral coping skills (yes/no), and the extent to which they used these skills within the past 4 weeks (not at all/a little/a lot). CBT: cognitive behavioral therapy. The skills were: identify thoughts or feelings I was having in response to my child’s cancer experience, remind myself that it is normal to feel this way after my child has finished cancer treatment, use different/new activities to interrupt a bad mood and give me a sense of balance, recognize unhelpful thoughts and how they are making me feel, use evidence for/against unhelpful thoughts to stop them from bothering me so much, recognize when I am going around in circles thinking about ‘what ifs’, ‘whys’, or questions that do not have an answer, reach out to my friends or family when I need them, raise difficult topics with people I am close to, set realistic personal goals, and figure out steps I can take to get there, plan how to deal with future situations that might bring up difficult thoughts or feelings.

There was evidence that at the post-booster time point (Q2), Cascade parents felt confident to use a higher number of CBT skills than both waitlisted parents (OR = 5.56, 95% CI = 1.81–17.12, *p* = 0.003) and peer-support group parents (OR = 3.06, 95% CI = 1.00–9.39, *p* = 0.05, Figure 4). There was no evidence of any difference between parents in the three groups in self-reported CBT skills use. See Figure 5.

##### Parents’ Mental Health Service Use in the Last Six Months

**Figure 6:**
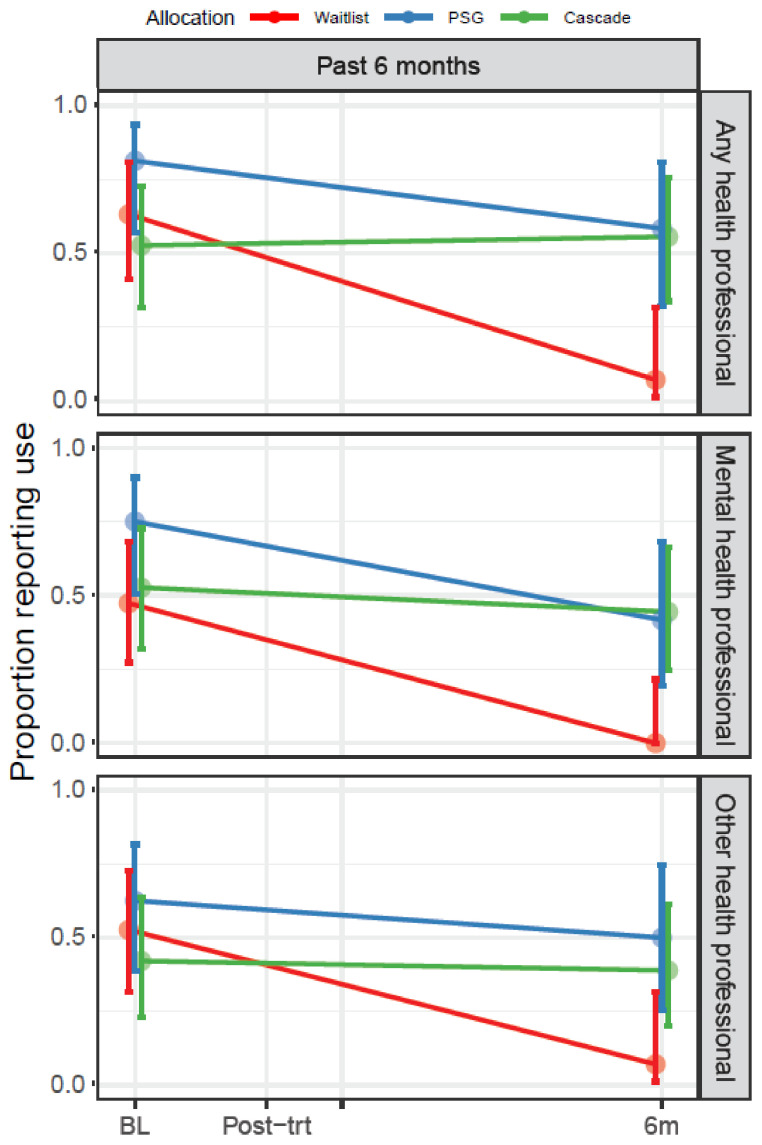
Proportion of parents who accessed any mental health professional or other health professional in the last six months, at baseline (Q1) and six months post-intervention (Q4). **Notes:** Mental health services included visiting a psychologist, social worker, counselor, or psychiatrist, as well as community-based cancer support organizations. Other health service use included visiting a general practitioner (family doctor), oncologist/radiation oncologist, nurse in hospital, or nurse in the community. BL: Baseline (Q1); Post-trt (Q2): After intervention; 6m: Six months after intervention (Q4).

Looking across all three groups, the proportion of parents who reported accessing support from a mental health professional at least once in the past six months declined over time. However, the proportion of parents accessing support declined most in waitlisted participants, relative to the parents in Cascade or the peer-support groups (*p* = 0.01; Figure 6).

##### Parents’ Other Health Service Use in the Last Six Months

Reflecting a similar pattern to mental health service use, looking across all groups, the proportion of parents who reported accessing support from other health professionals at least once in the past six months declined. However, the proportion of parents accessing support declined most in waitlisted participants relative to parents in Cascade or the peer-support groups (*p =* 0.02; Figure 6).

##### Parents’ Hospital Use in the Last Six Months

Sixteen-percent of parents (7/44) reported attending an emergency department for themselves in the last 6 months. Eleven-percent of parents reported being admitted to hospital in the last 6 months (5/44). There was no evidence of a difference between groups in the change between baseline (Q1) and at 6 months (Q4) in emergency department visits (*p* = 0.57) or hospital admissions (*p* = 0.29).

##### Parents’ Psychotropic Medication Use in the Last 4 Weeks

**Figure 7:**
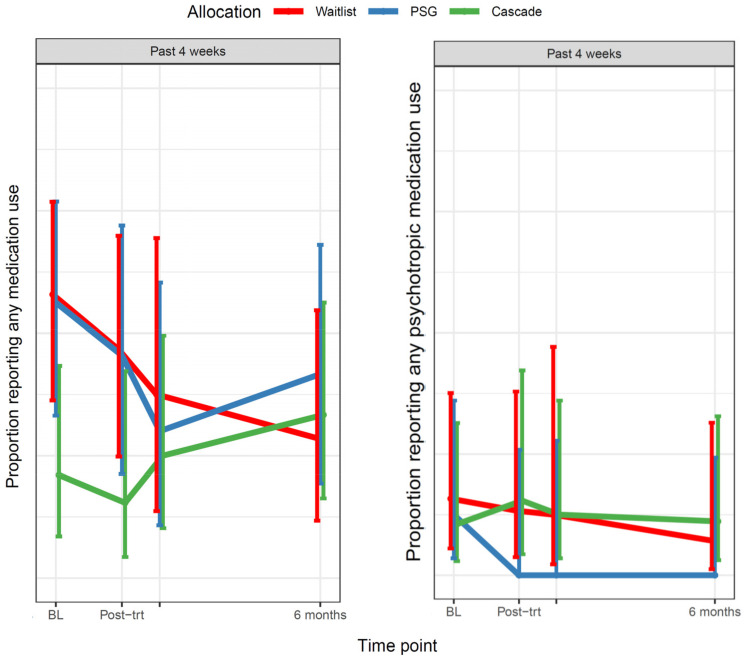
The proportion of parents reporting any medication use, and psychotropic medication use, in the last four weeks. Notes: Psychotropic medications included antidepressants, anxiolytics and mood stabilizers. BL: Baseline (Q1); Post-trt (Q2): After intervention.

At baseline (Q1), 11% of Cascade parents (2/19), 12% of peer-support group parents (2/16) and 16% of waitlisted parents (3/19) reported using psychotropic medication in the last 4 weeks. There was evidence of group differences in psychotropic medication use over time (*p* = 0.021): at six months post-intervention (Q4), no peer-support group parents reported using psychotropic medication in the last 4 weeks, compared to 7% of waitlisted parents (1/14) and 11% of Cascade parents (2/18). See Figure 7.

#### 2.12.4. Days Engaged with Productive Activities in the Last 4 Weeks 

**Figure 8:**
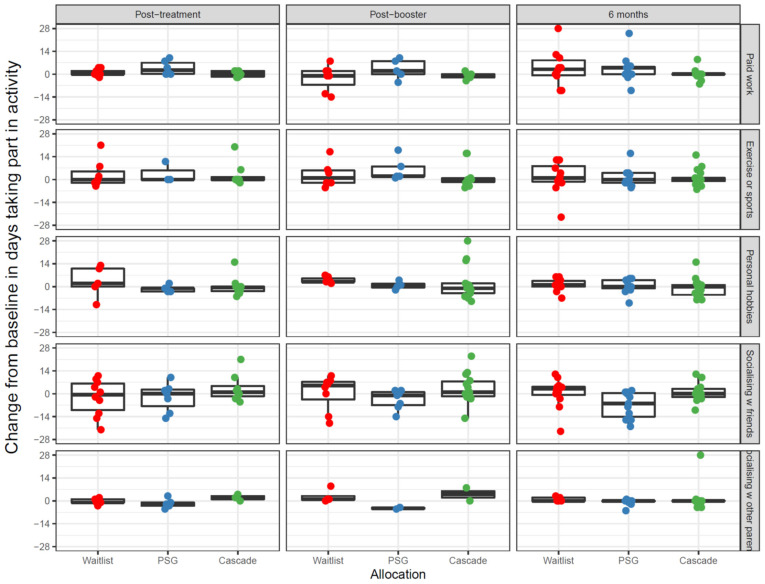
Change in the number of days parents engaged in productive activities in the last four weeks from baseline (Q1) at each time point for each parent. Notes: PSG: Peer-support group.

There was evidence that waitlisted and peer-support group parents typically reported small increases in paid work from baseline (Q1) to six months post-intervention (Q4) (3–4 days), while Cascade parents’ engagement in paid work was more constant (*p* = 0.02). For hobbies, waitlisted and peer-support group parents typically reported no change or a small increase in engagement in hobbies, while Cascade parents reported no change or a small decrease (*p* = 0.02). For socializing with friends, waitlisted parents typically reported a small increase, while Cascade parents reported no change or a small increase and peer-support group parents typically reported a decrease (*p* = 0.02). For socializing with other parents of children with cancer, waitlisted parents had slight increases on average, while peer-support group and Cascade parents typically stayed constant or reduced slightly (*p* = 0.03). Changes in parents’ engagement in exercise/sports did not appear to differ across the three groups (*p* = 0.56). See Figure 8.

## 3. Discussion

We evaluated a new videoconferencing group program for parents of childhood cancer survivors. The national trial did not meet our feasibility targets, although it did appear feasible to safely deliver the program across sites. Most parents who participated in Cascade reported being satisfied with the program and experienced benefits, although parents raised some concerns regarding the time commitment and feeling ill-prepared to share difficult experiences with other parents. Cascade parents did not report improvements in HRQL, anxiety, depression, communication, worry about their child’s health, daily activities, parenting self-agency, or fear of cancer recurrence, compared with peer-support group or waitlisted parents. There appeared to be no group differences in parents’ reports of their child survivors’ overall HRQL or in the physical health domain. However, Cascade parents reported lower scores for their child in the psychosocial health domain compared to waitlisted parents at six months and also did not experience improvements in their health service and psychotropic medication use or productive activities over time. Cascade parents reported feeling confident to use a higher number of CBT skills than waitlisted and peer-support group parents in the short term; however, this did not appear to translate to actual use of CBT skills.

### 3.1. Feasibility

Aligning with other studies in the field [23], we experienced significant challenges with recruitment. While we were not able to calculate response rates at each site, it was more difficult to recruit parents from hospitals other than the lead site: 34 parents from Sydney Children’s Hospital completed the baseline (Q1) questionnaire, versus only 22 parents across the remaining 7 hospitals. This uneven recruitment pattern may have been due to the fact that Sydney Children’s Hospital was the first to open the trial, allowing additional time for recruitment. It is also possible that parents at the lead site had a closer affiliation with the hospital leading this study (and so were more inclined to participate) and/or that there were fewer resources available to prioritize recruitment at the other hospitals. However, once parents expressed an interest in Cascade, it was feasible to deliver the program nationally. The enrolment rate (after opting in), attendance at sessions, and attrition from Cascade, were reasonable. We demonstrated that with careful planning [66], it is possible to safely deliver support to parents across the country using an online group program delivered from a central site. Confirming that the technology was already available to the vast majority to Australian families [67], almost all parents had the required equipment. While technological difficulties were common, most disruptions were minor. It is likely that since the COVID-19 pandemic, technology access and confidence has further improved which will hopefully further lower barriers to delivery in the future [68,69].

### 3.2. Acceptability

Most parents who participated in Cascade were satisfied, with all Q2 respondents indicating that they ‘enjoyed having other people to discuss issues with’. Most parents reported that Cascade was at least ‘quite a bit’ beneficial. Mirroring findings in face-face group programs for parents [70], parents valued feeling connected to others with similar life experiences. Cascade parents reported achieving a good level of cohesion with their other group members and good alliance with their psychologist. Few parents reported that the program was burdensome; however, their qualitative comments were valuable, particularly highlighting the time burden, which is a common help-seeking barrier [71]. Some parents also indicated that they felt unprepared to discuss difficult topics, or felt distressed by hearing others’ difficult stories. Some also expressed surprise that the program was developed for their personal therapeutic benefit, rather than being purely to support research. It may therefore be helpful in future programs to assess participants’ expectations and preferred types of treatment before they commence a group [72].

### 3.3. Efficacy

Despite parents desiring post-treatment support [4] and our extensive pilot-testing [34], Cascade did not significantly improve parents’ HRQL or other measured psychosocial outcomes. There are multiple possible explanations for the lack of positive findings. First, we may not have recruited sufficient participants to demonstrate an effect (target at Q2: 120 parents, achieved: 39), although the achieved confidence intervals did not appear to include a meaningful effect. Second, the lack of a positive finding may have been due to Cascade’s preventative approach, which did not restrict enrolment to parents with poor HRQL or high distress. Aligning with parents in other studies [23,72,73,74], many of our participating parents actually reported good HRQL and low depression/anxiety at baseline (Q1) and may have had a low need for further help, limiting the relative improvement that could be achieved. It is possible that a program delivered further off treatment, when survivors’ risk of late medical complications and challenges of developing independence into adulthood are increased, may be more effective. Long-term survivorship can indeed be a time when the full impact of the childhood cancer diagnosis is fully appreciated and additional supports at this time might be valuable [75].

Third, Cascade’s group format may not have been helpful for all parents. For some parents, aspects of their child’s cancer experience may have been traumatic and discussing this in a group setting may have increased their awareness of difficulties that they had not yet fully processed. This increased awareness may explain why Cascade parents perceived that their child’s psychosocial health was lower than waitlisted parents and may explain our findings that Cascade parents’ health service use, psychotropic medication use and days engaged in productive activities did not reduce, despite reductions being observed in waitlisted parents. While many people affected by cancer desire connections with others, group support programs do not suit everyone. A recent meta-analysis of psychological interventions for parents of children with cancer reported that group programs were less effective than individual programs, possibly because participants in individual programs have more opportunities for communication and they can receive more personalized feedback [27]. A recent exploration of parents’ intervention preferences also reported that parents expressed a strong desire for being offered a choice between participating in a group or an individual program. Some parents in that study expressed a strong desire to avoid ‘forced’ group interaction, with some explicitly stating that they would not attend a group program if that was the only choice [76]. It is therefore possible that Cascade’s group format may have influenced both the response rate (with some parents avoiding the group program upfront), but also Cascade’s actual impact (with some parents finding that the group format was not as helpful as they anticipated). Future research exploring the factors that influence parents’ preferences for group versus individual support programs would be valuable.

Fourth, while our findings regarding Cascade parents feeling more confident to use a higher number of CBT skills than those in other arms were promising, these effects were not maintained after six months and did not generalize to self-reported actual use of CBT skills. We did increase the ‘dose’ of the program from three to four modules after the pilot; however, it is possible that Cascade still did not provide a sufficient ‘dose’ of therapy to achieve translation into action, with typical CBT program length being 12+ sessions [77]. Given the typical length of CBT programs, it is possible that our findings regarding parents’ health service use and medication use could actually be interpreted as an indicator of intervention success, with participants in Cascade and the peer-support groups continuing to use services and medications that they needed, while waitlisted participants discontinued use early. Increasing ‘dose’ by adding extra modules/boosters or by increasing the amount of experiential practice of key skills, has been recommended by other intervention developers [76] and might have increased Cascade’s potency; however, time was already the most cited burden of participation. Achieving the right balance between ‘dose’ and ‘burden’ will be key for future studies [78]. Despite strong evidence that CBT can be helpful for parents [15,19,30], it is also possible that CBT was not the ideal therapeutic approach with which to tackle parents’ concerns, with other therapeutic approaches also potentially adding value to parents at this timepoint (e.g., acceptance and commitment therapy [22]). It is also possible that focusing on only one family member was less efficacious than taking a couples, or family, approach to intervention [31,79].

Finally, the lack of significant findings may have been because we focused on measurement of HRQL, which is a relatively distal outcome (although we did include several other outcome measures as well). Nonetheless, it may have been more feasible to improve other outcomes that map more closely to the content of Cascade, such as perceived peer-support, emotion regulation, or resilience [27,28]. There is also evidence that it is not uncommon for trials of group programs in cancer to find that perceived benefits are difficult to capture with quantitative measures, even if qualitative feedback is positive [33]. Any benefits might also be difficult to capture if they are short-lived or only experienced by some participants, rather than the whole group [33]. It is also possible that our choice of an attention control (a peer-support group covering the same topics as Cascade) made it more difficult to detect changes between the two intervention groups (i.e., Cascade versus the peer-support group)- indeed it is possible that the peer-support group was a powerful intervention itself. However, both interventions did not yield long-term positive outcomes relative to the waitlist.

### 3.4. Study Strengths and Limitations

This study had several strengths. Rigorous trials are rare in pediatric psycho-oncology [80]. Most studies in this area do not include a control group or any longer-term follow-up [33]. Our inclusion of an attention control (the peer-support group) was novel and aligned with recent calls in the field [81]. The trial took longer than anticipated to conduct and did not achieved the final target sample size, despite including multiple sites. We made multiple amendments to the trial design in attempt to increase recruitment, with limited success. The effective sample size for analysis was small, about a third of the target sample size, and was thus underpowered for the effect size specified during trial development. We conducted multiple comparisons with a small sample, hence inflating our risk of Type I (false-positive) errors, so some of the statistically significant findings may be due only to the play of chance. We were not able to collect data on non-respondents and we recruited few fathers. Given the small sample size for fathers, we did not account for clustering within families. The group approach meant that all parents needed to be English-speaking. Psychosocial and peer-supports accessed by parents outside of the program might have influenced the impact of Cascade, with some parents possibly receiving pre-existing supports from community-based psychologists or through their general practitioner [72]. Future research examining the impact of the program in families affected by different types of cancers (e.g., leukemias versus brain tumors) would also be valuable, given that different cancer types result in different risks of recurrence and different medical late complications for survivors.

### 3.5. Future Directions

Based on our learnings in this trial, our team, in conjunction with several child and adolescent cancer community organizations (including Redkite and Canteen Australia), has now developed a modified Cascade program, which will be further evaluated through these organizations. We have improved our introductory materials to ensure that parents who opt-in fully understand the program goals. Program adaptations include offering an additional introductory module for parents to share their stories and allow development of shared understanding between group members before delivery of the core content. We have also modified the program manual such that the expertise of parents is further reinforced, for example by inviting parents to use their own metaphors, allowing more time in session to discuss positive changes after cancer and reducing the focus on fear of the cancer returning (allowing parents to raise this more sensitive topic when they feel ready). Perhaps most importantly, we have adapted the program to allow parents to choose to participate in the program individually (with one-to-one delivery of the materials from the psychologist), or in a group if they would prefer. We will carefully monitor the logistical and cost/resource implications of these changes. We have also modified our evaluation design, such that we will also assess emotion regulation, coping self-efficacy and peer-support, which map well to the content of Cascade and CBT more broadly.

We have also used the feasibility data collected in this trial to simplify our next trial design and develop more realistic estimates of the likely number of parents who will opt-in to Cascade each year. Indeed, our data are valuable for future research triallists. Even with multiple amendments to our original trial protocol [35], our trial did not meet our recruitment targets, with slower than anticipated launch of the trial at each site, and lower than anticipated participant accrual. Mirroring our experiences with other similar studies [82], launching the trial at eight sites (including securing separate ethical/governance approvals for each hospital) took longer than anticipated. Even post-approval, many parents did not respond to study invitations and it took longer than planned to form each group. Future research should explore ways to use more feasible trial designs that are more appealing to potential participants. Trialists could consider streamlining data collection to minimize participant burden and could consider using more innovative methods to improve participation (e.g., video invitations that provide clear information about what to expect and the time commitment involved) [83,84]. Future intervention deliverers, including community organizations looking to broaden their reach to families online, should plan carefully for the fact that the program will likely appeal to a relatively small group of parents, that groups may take several weeks to form, and that many parents will require the program to be delivered out of business hours. Parents appeared to be willing to complete the home practice activities, but it might also be helpful to offer more support/reminders to increase completion (also requested by parents in a similar study [76]).

## 4. Conclusions

This trial evaluated a group videoconferencing intervention for parents of childhood cancer survivors. The trial design was difficult to implement and resulted in missing its recruitment targets. Once parents did opt-in to the program, it was feasible to deliver Cascade nationally, although the program required out-of-hours delivery for almost half of the sessions. Cascade participants were satisfied with the program, but some reported burdens, particularly regarding the time commitment and the group format. Cascade was not able to achieve improvements in the primary and secondary outcomes, and although it did achieve a short-term improvement in the number of CBT skills parents felt confident to use, this did not eventuate in actual use of these skills. It is likely that while some parents will find Cascade helpful, the program will not meet the needs of all parents after the completion of their child’s cancer treatment. Given the small number of rigorous trials in this area, these findings contribute to increasing our understanding of the factors that may influence parents’ participation and responses to psychological approaches to cancer-related distress and therefore contribute important new data to this growing field.

## Figures and Tables

**Table 1:** Key content within Cascade and underlying cognitive behavior therapy components ^a^.

Module	Cascade Program Content	Cognitive Behavior Therapy Components
1: ***‘What just happened to us?’*** Introduction and behavioral activation	Introduce the ‘ship in the storm’ metaphor Discuss impact of having a child with cancer on family activities and routines. Reflect on any changes and impacts on stress and mood using the Achievement, Connectedness, Enjoyment (ACE) model. Identify activities to help regain balance (if needed). Monitor activities and experiment with increasing ACE activities	Program engagement Rationale for learning skills Normalizing common parent experiences/behavioral responses to their child’s cancer/treatment Behavioral activation
2: ***How has cancer changed the way I think?*** Identifying and challenging unhelpful thoughts	Identify concerns or worries participants have experienced since their child finished treatment Apply the cognitive model to examples (e.g., child comes home from school with a headache) Discuss thinking styles and the role they can play in maintaining and amplifying distress, including examples specific to childhood cancer (e.g., Shoulds and Musts; *“I shouldn’t still be upset about my child having had cancer because they are better now”*) Discuss rationale for identifying and challenging unhelpful thoughts, including identifying types of thoughts amenable to challenging. Discuss how to challenge unhelpful thoughts	Brief psychoeducation about the cognitive model Normalizing distressing thoughts and worries Thought monitoring Recognizing unhelpful thinking styles Thought challenging to manage unhelpful thoughts
3: ***Out of your head and back into your life*** Mindfulness and disengagement	Facilitate discussion about ‘big’ existential thoughts that parents can have after their child’s treatment (e.g., fear of recurrence, death and dying) Experiment with the ‘polar bear’ experiential exercise to demonstrate the futility of thought suppression Build on cognitive challenging to discuss other ways participants can respond to unhelpful thoughts that cannot be easily challenged (e.g., ‘*What if my child’s cancer comes back?’and ‘Why did this happen to our family?’*) Rationale for, and overview of responding to unhelpful thoughts with acceptance/disengagement and mindfulness	Identifying and normalizing existential concerns that parents commonly report following their child’s treatment Psychoeducation about thought suppression Identifying thoughts that are difficult to challenge (e.g., rumination, existential worries) Psychoeducation about strategies for accepting and letting go of unhelpful thoughts that are difficult to challenge (acceptance and redirecting attention, mindfulness)
4: ***Looking forward*** Skills for fostering relationships and living a rich life after cancer	Discussion of parents’ experience of social support during their child’s cancer experience Identify difficulties in relationships and communication ‘traps’ and discuss how to apply assertiveness skills and cognitive challenging Revisit the ‘ship in the storm’ metaphor to reflect on current situation and discuss goal setting	Normalizing changes in relationships Rationale for importance of social support Psychoeducation about assertive communication, including assertive communication skills Applying mindfulness to relationships Goal setting/values clarification and psychoeducation about psychological relapse prevention (i.e., how to revisit skills when new psychological challenges emerge in the future)
Booster session	Discussion of participants’ progress in light of goal(s) identified at the start of the program and the skills covered Discussion of ongoing implementation of skills learned and support needs	Review progress made against goals identified Review ongoing application of skills Review ongoing needs

^a^ Cascade is a synchronous online intervention, delivered live (in real time) by a psychologist using videoconferencing with 2–5 parents/group. Parents received a psychoeducational workbook to supplement the modules. Four weeks after the program, parents received a one-hour one-to-one booster session with the psychologist who delivered their intervention to consolidate skills learnt and discuss any challenges.

**Table 2:** Measures used to assess Cascade and the Cascade trial.

Domain	Outcome Measure	Details
Feasibility of the national trial		
Timeliness	Length of trial: Time taken to recruit sufficient participant numbers	We assessed the number of months from the study opening at the first site to the study close out date. Original recruitment target was 15 months.
	Data flow-through: Data systematically recorded in study files	We assessed time from baseline questionnaire (Q1) completion to commencement of Cascade or the peer-support group in days.
Trial participation	Trial response rate: Proportion of participants opting in to the trial	Total number of trial opt ins divided by the total number of potential participants provided with study information [only able to be calculated at one site, Sydney Children’s Hospital] (**target > 40%**).
	Trial enrolment rate: Proportion of participants who moved through to randomization after opting in to the trial	Total number of randomized parents divided by the total number of parents who opted in (target not pre-specified).
	Trial attrition rate: Proportion of participants who did not complete their final questionnaire	Total number of baseline (Q1) participants–number of 6 month follow-up questionnaire completions divided by the total number of baseline participants (**target < 20%**).
**Feasibility of delivering the Cascade intervention**		
Treatment fidelity	Independent ratings of the adherence of the content to the Cascade manual	Independent raters (blind to allocated arm) watched > 15% of randomly selected sessions. Raters then indicated which arm the session belonged to and how adequately key Cascade treatment components were covered on a 7-point scale (0 = not covered, 1 = marginal, 4 = acceptable, 6 = high).
Participant engagement	Session engagement (total sessions attended/parent)	Psychologists recorded every session attended by each parent.
	Session length	Recorded by psychologists at every session and confirmed by research officers.
	Engagement in home practice activities set by Cascade	Cascade parents reported the amount of home practice activities they completed after each module (‘none’, ‘some’, ‘all’). This information was not shared with the psychologist running each Cascade session to avoid parent desirability reporting bias. Adapted from the *Homework Compliance Scale* [43].
Reach	Proportion of participants participating from regional/remote locations	We recorded participants’ home postcode in order to categorize their residential area as urban versus regional/remote.
	Number of sessions delivered to participants in different states	We recorded the state from which each participant joined each session. Session length was calculated as the difference between actual start time and end time in minutes.
Organizational impacts	Proportion of sessions that occurred outside business hours	Data systematically recorded in study files. We considered any session start or end time before 9am or after 5pm as ‘out of business hours’).
Technological difficulties	Time for session to commence	Data systematically recorded in study files. Time for session to commence was calculated as the difference between the scheduled start time and time the last group member joined in minutes.
	Loaned equipment	We assessed the number of participants who needed to borrow technology to participate.
	Technological issues	The psychologist recorded all technological issues that occurred during each session (categorized as ‘audio’/‘visual’/‘login’/‘other’) and the perceived disruptiveness of the issue (1 = ‘not at all disruptive’ to 10 = ‘completely disrupted session’).
**Acceptability**		
Intervention satisfaction; Q2 Cascade parents only	Adapted satisfaction questionnaire [44]	The adapted satisfaction questions included 10 items assessing parents’ perceptions of the key components of Cascade, including the usability of the online format, relevance of the modules to their own child cancer experience, the amount and quality of information provided by Cascade, new skills learnt in Cascade, relevance of the skills learnt, usefulness of skills learnt, helpfulness of talking about new skills, enjoyment in discussing issues with other parents in the group, helpfulness of the home practice activities and the helpfulness of the Cascade workbook. (0 = ‘strongly disagree’ to 5 = ‘strongly agree’).
Perceived benefit and burden; Q2 only	*Purposely developed and previously tested in a cancer sample* [45]	We collected parents’ ratings regarding whether Cascade/peer-support group was beneficial/burdensome in any way (0 = ‘not at all’, 1 = ‘somewhat’, 2 = ‘quite a bit’, to 4 = ‘very much’), with an open-ended question inviting further elaboration.
Group cohesion	*California Psychotherapy Alliance Scale for Groups* (CALPAS-G) [46]: administered after each Cascade and peer-support group module	Following each session, Cascade and peer-support group participants rated the perceived openness, acceptance, appropriateness, and understanding in their group using four items from the CALPAS-G (1 = ‘not at all’ to 7 = ‘very much so’). We calculated an overall group cohesion score by averaging all responses.
Working alliance	*Working Alliance Inventory* (WAI) [47]: administered after each Cascade and peer-support group module	Cascade and peer-support group members completed four items from the WAI after their first and last modules to rate participants’ perceptions of their working alliance and bond with their facilitator (1 = ‘doesn’t correspond at all’ to 7 = ‘corresponds exactly’).
**Psychological safety of Cascade**		
Emotional distress and need for help	*Emotion Thermometers Tool* [48]: administered after each Cascade and peer-support group module	Between each Cascade/peer-support group session, parents rated their emotions in the past week (distress, anxiety, depression, anger) and their need for help on a scale of 0 = ‘no distress’ to 10 = ‘high/extreme distress’ on a visual ‘thermometer’. Based on the clinical consensus of the multidisciplinary investigator team (oncologists/psychologists/nurses/social workers) [45], we defined a ‘clinically concerning occasion’ as reporting a score of ≥7 on any thermometer or an increase of >3 points from the previous score.
**Efficacy**		
Health-related quality of life (HR-HRQL) [primary outcome]; Q1–Q5	*PedsQL Family Impact Module* [49]	We assessed HRQL using 18 items from the physical, emotional, social and cognitive functioning scales of the PedsQL Family Impact Module [49], The PedsQL uses a 5-point response scale (0 = ‘never a problem’ to 4 = ‘always a problem’). The validated summary HRQL score is computed as the sum of the items divided by the number of items, where higher scores indicate better functioning (i.e., less negative impact). Cronbach’s alpha for this study, 18 items: 0.95 (0.94, 0.95).
	*6-item EQ-5D-5L (including the EQ Visual Analogue Scale [EQ-VAS])* [50]	The EQ-5D-5L assesses mobility, self-care, ability to participate in usual activities, pain/discomfort, anxiety/depression, and overall perceived health. Using Norman et al.’s (2013) Model D [51], we converted EQ-5D-5L responses into a quality-adjusted life-year weight, where 1.0 represents full health. The EQ-VAS accompanies the EQ-5D-5L, where parents self-rate their health on a visual analogue scale (100 = ‘the best health you can imagine’ to 0 = ‘the worst health you can imagine’).
Parents’ psychological outcomes; Q1–Q5	Communication, worry about child’s health, daily activities and family relationships assessed with *PedsQL Family Impact Module* [49]	The PedsQL Family Impact Module includes an additional four subscales which are not included in the HRQL summary score. These subscales assess how much of a problem participants have had with communication (3 items), worry about the child’s health and its impacts (5 items), daily activities (3 items) and family relationships (5 items). The subscales use a 5-point response scale (0 = ‘never a problem’ to 4 = ‘always a problem’). Cronbach’s alpha for this study, 3-item communication subscale: 0.82 (0.77, 0.87); Cronbach’s alpha, 5-item worry subscale: 0.85 (0.92, 0.88); Cronbach’s alpha, 3-item daily activities subscale: 0.91 (0.89, 0.94); Cronbach’s alpha, 3-item family relationships subscale: 0.95 (0.94, 0.96).
	Anxiety and depression assessed with *PROMIS Anxiety Short Form PROMIS Depression Short Form* [52]	We assessed anxiety using the 7-item PROMIS Anxiety Short Form and depression using the 8-item PROMIS Depression Short Form [52]. Higher scores represent worse anxiety/depression (1 = ‘never’ to 5 = ‘always’). Cronbach’s alpha for this study, 7-item anxiety subscale: 0.94 (0.93, 0.95); Cronbach’s alpha, 8-item depression subscale: 0.92 (0.91, 0.93).
	Parenting self-agency assessed with the *Parent Self-Agency Measure (Revised) (PSAM-R)* [53]	We assessed parents’ level of confidence in their ability to engage in successful parenting behaviors via the 5-item PSAM-R (1 = ‘rarely’ to 7 = ‘always’). Cronbach’s alpha for this study, 5 items: 0.80 (0.75, 0.83).
	Fear of cancer recurrence: *Fear of Cancer Recurrence Inventory* (FCRI) [54]	Parents reported their fear of recurrence in their child using three items from the 3-item severity subscale of the FCRI, assessing perceived risk (‘not at all’ to ‘a great deal of risk’, worry (‘not at all’ to ‘a great deal’, and frequency of thinking about recurrence (‘never’ to ‘everyday’). Cronbach’s alpha for this study, 3 items: 0.77 (0.70, 0.83).
Confidence to use, and actual use of, CBT skills; Q1–Q5	*Purposely developed and previously tested in a cancer sample* [45]	We used 10 purposely developed items to assess parents’ confidence to use a series of cognitive and/or behavioral coping skills (yes/no), and the extent to which had used these skills within the past 4 weeks (not at all/a little/a lot).
Parents’ perceptions of their child’s quality of life; Q1–Q5	*PedsQL Generic Core Module–parent proxy report* [55]	23 items assessing the child’s quality of life, including the child’s physical, emotional, social and school functioning. PedsQL is designed to provide greater measurement sensitivity to patient populations and is widely used in cancer [56]. Cronbach’s alpha for this study, 20 items total score *: 0.93 (0.92, 0.93); Cronbach’s alpha for this study, 8 items physical health subscale *: 0.93 (0.92, 0.94); Cronbach’s alpha for this study, 12 items psychosocial health subscale *: 0.87 (0.85, 0.88)._ENREF_64
Health and mental health service use in the last six months; Q1 and Q4	*Items based on those used in a previous study* [57]	Mental health services included visiting a psychologist, social worker, counselor, or psychiatrist, as well as community-based cancer support organizations. Other health service use included visiting a general practitioner (family doctor), oncologist/radiation oncologist, nurse in hospital, or nurse in the community.
General functioning; Q1–Q5	Indexed by time spent engaging in productive activities and days absent from work over the past four weeks. *Items based on those used in a previous study* [57]	Parents indicated the number of days they spent engaging in productive activities and days absent from work in the last four weeks. Productive activities included ‘Paid work of any kind’, ‘Exercise or sports’, and ‘Personal hobbies’ (e.g., art, music, films, books, outdoor activities, cooking)’, ‘Socializing with friends’, and ‘Socializing with other parents of child cancer survivors (including connecting online)’.

* Questions differed depending on age group, with the biggest difference being that 2–4 year olds were only asked 3 questions about (pre)school whereas older children were asked 5. We therefore excluded the non-matching questions in calculating Cronbach’s alpha values, rather than excluding 2–4 year olds, who formed 31% of the children represented in this study.

**Table 3:** Baseline (Q1) sociodemographic characteristics of parents and clinical characteristics of their child who survived cancer.

Characteristic	Total Sample (*n* = 56)	Cascade (*n* = 19)	Peer-Support Group (18)	Waitlist (*n* = 19)
Participants’ recruiting site: *n* (%) (*n* = 56 ^a^)				
Children’s Hospital Westmead (NSW)	4 (7)	0 (0)	4 (22)	0 (0)
John Hunter Children’s Hospital (NSW)	1 (2)	0 (0)	0 (0)	1 (5)
Queensland Children’s Hospital (QLD)	4 (7)	2 (11)	1 (6)	1 (5)
Monash Children’s Hospital (VIC)	1 (2)	0 (0)	0 (0)	1 (5)
Princess Margaret Hospital (WA)	4 (7)	0 (0)	0 (0)	4 (21)
Royal Children’s Hospital (VIC)	7 (12)	2 (11)	0 (0)	5 (26)
Sydney Children’s Hospital (NSW)	34 (61)	15 (79)	12 (67)	7 (37)
Women’s and Children’s Hospital (SA)	1 (2)	0 (0)	1 (6)	0 (0)
Gender: *n* (%) (*n* = 56)	Female: 49 (88%)	Female: 17 (89%)	Female: 16 (89%)	Female: 16 (84%)
Age: median (IQR, range) (*n* = 53)	40 years (38–45, 20–55)	41 years (39–45, 30–49)	41 years (37–45, 20–55)	40 years (38–44, 31–49)
Residence: *n* (%) (*n* = 55)	Major city: 47 (85%) Inner regional: 8 (15%)	Major city: 14 (74%) Inner regional: 5 (26%)	Major city: 16 (94%) Inner regional: 1 (6%)	Major city: 17 (89%) Inner regional: 2 (11%)
Distance from state capital: median (IQR, range) (*n* = 55)	29 km ^b^ (12–102, 5–811)	30 km (10–180, 5–518)	59 km (12–292, 7–572)	25 km (13–49, 6–811)
Age of the child who had cancer: median (IQR, range) (*n* = 53)	7 years (4–11, 2–17)	7 years (6–10, 2–15)	11 years (4–15, 2–16)	6 years (4–7, 2–17)
Diagnosis category: *n* (%) (*n* = 54)	Blood cancer: 21 (38%) Brain cancer: 11 (20%) Other solid tumor: 21 (38%)	Blood cancer: 8 (42%) Brain cancer: 2 (11%) Other solid tumor: 8 (42%)	Blood cancer: 4 (24%) Brain cancer: 4 (24%) Other solid tumor: 8 (47%)	Blood cancer: 9 (47%) Brain cancer: 5 (26%) Other solid tumor: 5 (26%)
Time since treatment completion: median (IQR, range) (*n* = 52)	14 months (9–21, 4–129)	14 months (11–19, 4–32)	13 months (10–20, 4–37)	15 months (6–57, 4–129)
Previous relapse: *n* (%) (*n* = 54)	Yes: 9 (17%) No: 43 (81%) Don’t know: 1 (2%)	Yes: 2 (11%) No: 16 (89%) Don’t know: 0 (0.0%)	Yes: 4 (25%) No: 12 (75%) Don’t know: 0 (0%)	Yes: 3 (16%) No: 15 (79%) Don’t know: 1 (5%)

^a^ *n* provided for each variable to indicate any missing data; ^b^ 1 km = 0.62 miles. NSW: New South Wales, QLD: Queensland, VIC: Victoria, and SA: South Australia.

**Table 4:** Feasibility and acceptability indices within the Cascade trial.

**Organizational impacts**	Staff time commitment to deliver groups	We delivered 34.5 hours’ worth of Cascade content (median session time = 91 min, range = 60–115). Peer-support group sessions were delivered over 35 h (median session time = 88 min, range = 60–104).
	Staff time commitment to deliver booster sessions	We provided 15 one-to-one booster sessions for Cascade participants (totalling 6.3 h) and 3 group booster sessions for peer-support group participants, totalling 2.6 h.
	Working hours	Of the 47 group sessions delivered, we conducted 23 sessions (49%) out of business hours.
**Timeliness**	Group commencement	Median wait time from Q1 completion to group commencement was 35 days (range = 1–211)
	Median wait time to session commencement	3 min (range = 0–50).
**Technological considerations**	Access to equipment	Almost all parents (92%, 48/52 ^a^) had access to the required technology (web-enabled device with microphone/camera).
	Technical difficulties	Psychologists recorded at least one technical difficulty in 66% of sessions (31/47), most commonly poor-quality audio (16/47, 34%). For the 31 sessions in which at least one technical difficulty occurred, the median psychologist-rated disruptiveness score was 2 out of 10 (SD = 1.3, range = 1–7).
**Participant engagement**	Percent of sessions completed by participants	Most participants attended at least three sessions (Cascade: 17/18, 94%; peer-support group: 17/20, 85%). Most parents attended all four sessions (Cascade: 13/18, 72%; peer-support group: 16/20, 80%).
**Perceived benefit**	Cascade	Most parents reported that Cascade was ‘quite a bit’ to ‘very’ beneficial (9/13, 69%).
	Peer-support group	Most peer-support group parents rated the peer-support group as ‘quite a bit’ to ‘very’ beneficial (9/15, 60%)
**Perceived burden**	Cascade	Almost all Cascade parents reported that participation was ‘not at all’ to ‘a little bit’ burdensome (12/13, 92%).
	Peer-support group	Most peer-support group participants rated it as ‘not at all’ to ‘a little bit’ burdensome (13/15, 87%).

^a^ *n* provided for each variable to indicate any missing data.

## Data Availability

The data presented in this study are available upon reasonable request from the corresponding author, as is the full study protocol and the intervention materials. The data are not publicly available due to restrictions within the ethical approval.

## Supplementary Materials

The following are available online at https://www.mdpi.com/article/10.3390/cancers13225597/s1, Table S1: Summary of study design challenges, protocol modifications to address challenges and outcome of the modification, Table S2: Summary of parents’ responses to open-ended questions about the perceived benefits and burdens of Cascade, Figure S1: Parent attendance in Cascade and peer-support groups, by session, Figure S2: Overall group cohesion ratings for both Cascade and the peer-support groups post-intervention, Figure S3: Working Alliance Inventory ratings for both Cascade and the peer-support groups at week 4 (conclusion of the programs), Figure S4: Emotion Thermometer ratings for Cascade and peer-support groups across each time-point.
