# Supplementary material for: Providing Psychological Support to Parents of Childhood Cancer Survivors: ‘Cascade’ Intervention Trial Results and Lessons for the Future
Source: Cancers (Basel). 2021 Nov 9;13(22):5597. doi: 10.3390/cancers13225597 (PMC8615912; doi:10.3390/cancers13225597)
Supplement: Supplementary file 1 [file cancers-13-05597-s001.zip › cancers-1419152-supplementary.pdf]

Supplementary Table S1. Summary of study design challenges, protocol modifications to address challenges and outcome of the modification

Study design challenge	Protocol modifications to address identified challenges	Outcome of the modification
Difficulties recruiting sufficient participants	Inclusion of additional three recruiting hospitals (modification from five to eight sites)	Small increase in participants (total of 6 additional participants from the 3 hospitals), but did not reach target sample size
	Advertisement of the study through online social media platforms of relevant organizations (modification to add social media advertisements to study)	Yielded some interest in the trial, but did not appear to increase enrolments
Some invited parents reported it was too soon post-cancer treatment to participate	Extension of eligibility criteria (modification from parents who had a child who had completed cancer treatment with curative intent in the last 12 months to past 10 years)	Participation of parents across the 10-year post treatment completion period
Some parents of older children expressed interest in participating in the study	Extension of eligibility criteria (modification from parents who had a child currently aged <16 years to currently aged <18 years)	Participation of parents of 16- and 17-year-old children
Unexpectedly long delays to form groups	Original plan to stratify groups by rurality and severity of parents' distress amended to instead run mixed groups with regards to rurality and parent distress	Groups were successfully run with participants with differing characteristics, resulting in shorter delays
	Original plan to conduct recruitment in 12-week blocks was amended such that participants were assigned to	

	their allocated group, which was delivered once sufficient numbers for that group were achieved	
Few parents gave consent for their children to participate and few children completed questionnaires	Amended protocol to remove self-reported outcomes from children of participating parents	No available self-report data from child survivors or siblings
Complexities with randomization plan	Consensus from study team agreed that the amended original plan to use a flexible biased urn randomization was too complex for this study	Successfully implemented random order of groups delivered

Supplementary Table S2: Summary of parents' responses to open-ended questions about the perceived benefits and burdens of Cascade

Please tell us why Cascade was beneficial to you	*Please tell us why Cascade was burdensome to you*
General benefits:<br>• A brilliant and supportive program that addresses the isolation and worries that arise in the post-treatment phase of a cancer diagnosis.<br>• I really appreciated the chance to be a part of this study and to hopefully affirm the need for ongoing emotional support for parents in our situations. 4 weeks flew and I wished it was longer!<br>• I found this study was very meaningful to me and it will be something I will reflect on regularly.<br>• It was beneficial in the fact that it was convenient that I could do it from home.<br>• I was able to get some help with things that I didn't think I had the time to bother with.	***Time constraints:***<br>• I work full time so sometimes time was a constraint. However, the benefits definitely outweighed the burdens.<br>• The time out when you have two small children was sometimes difficult to manage.<br>• Two hours on a Monday night right at the time when I have to be feeding and bathing and putting my children to bed is quite challenging.<br>• The time of the meetings was in the middle of dinner time, but I worked around it.<br>• It was difficult to slot in 1.5hrs on a Monday night.
Connections with others and normalization:<br>• I really enjoyed seeing the other members in the group on a regular basis. I was interested in all of their experiences and felt that my concerns were validated by sharing both mine and their stories.<br>• I worried about one of the members in particular and her struggles, but saw the change in her over time and felt like I had seen something shift for her. It was nice to be a part of that.	***Reminders of difficult experiences:***<br>• It is over two years since she finished the worst of the treatment and I just want to put it all behind me rather than spending 2 hrs/week talking about it.<br>• Simply because it has been a while since my son finished treatment I felt it was very stressful after week 1 of Cascade. I think this was because going through the initial parts of diagnosis etc and talking about it all again opened up all the feelings again.

• It was beneficial to see other parents have similar life processes and issues.<br>• Participation in this study helped me realise that the feelings I were having were normal and appropriate reactions to the experience I had been through.	• Following the second session I found myself feeling quite anxious about my son's cancer. To put it simply, talking in an open forum made me realise its effect on me was still very raw. I still hadn't fully come to terms with it. I found myself feeling anxious about sharing the experience given I didn't feel balanced....in short, my can of worms had reopened and I wasn't prepared.
Skill development:<br>• Many thanks for giving me the opportunity to participate. I have certainly gathered valuable new skills that will help as I continue the quest of navigating this extraordinary and often relentless "journey"!<br>• Cascade provided me with insight, reassurance, and equipped me with some very useful skills that I can use going forward.	***Participating for research, not therapy***<br>• I wasn't looking for therapy of any kind, I agreed to do it because I thought I would be helping with research.<br>• It is therapy not research. Which is great, if that is what you are looking for.<br>• I'm not a huge fan of CBT as I think it can mask core issues - rather than unpacking and looking at why something might be causing distress/anxiety.

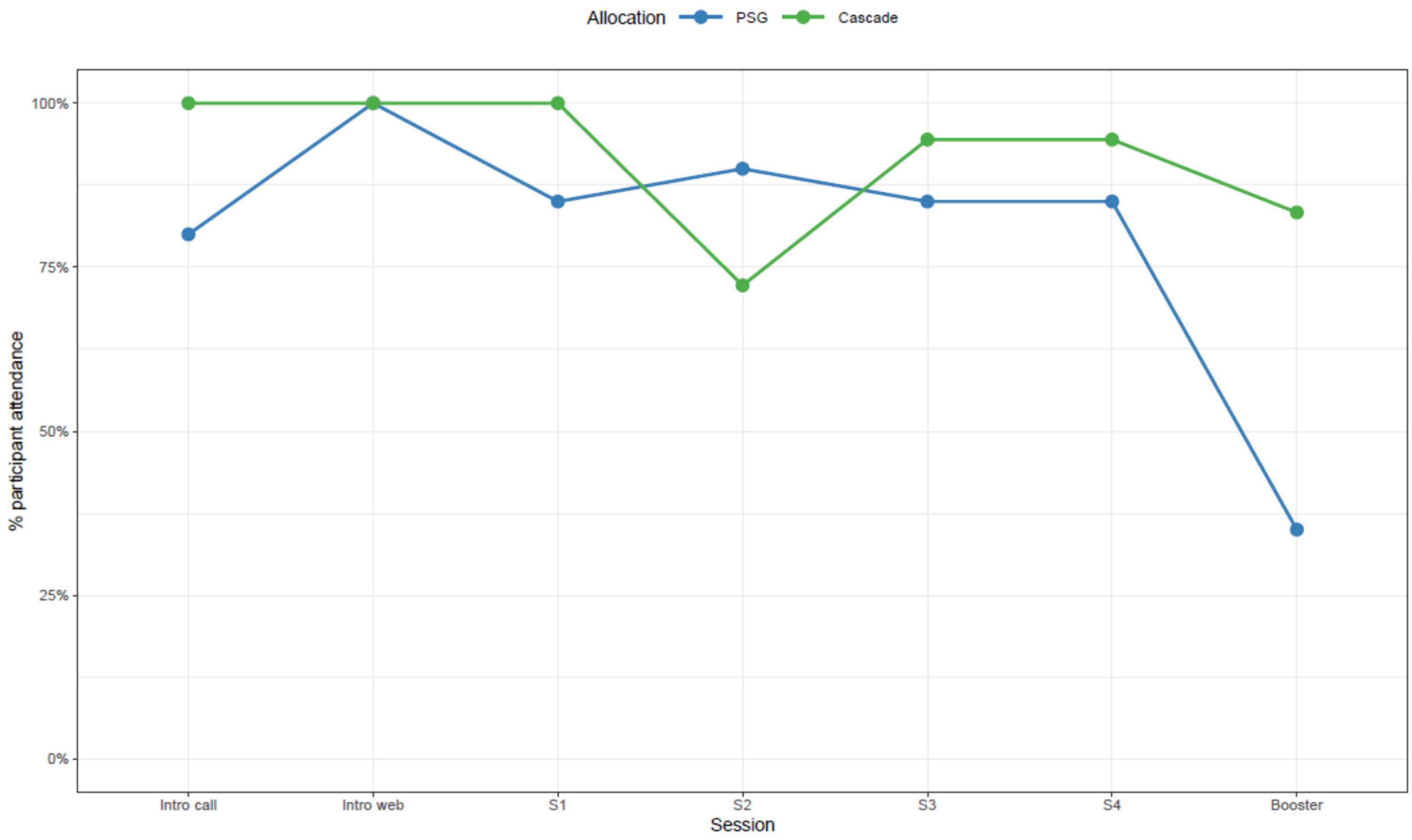

Supplementary Figure S1. Parent attendance in Cascade and peer-support groups, by session

Notes: PSG: Peer-support group; intro call: introductory phone call from psychologist; intro web: introductory phone call from research officer to check participants' access to required equipment and provide education about the online platform; S1-S4: sessions 1 to 4.

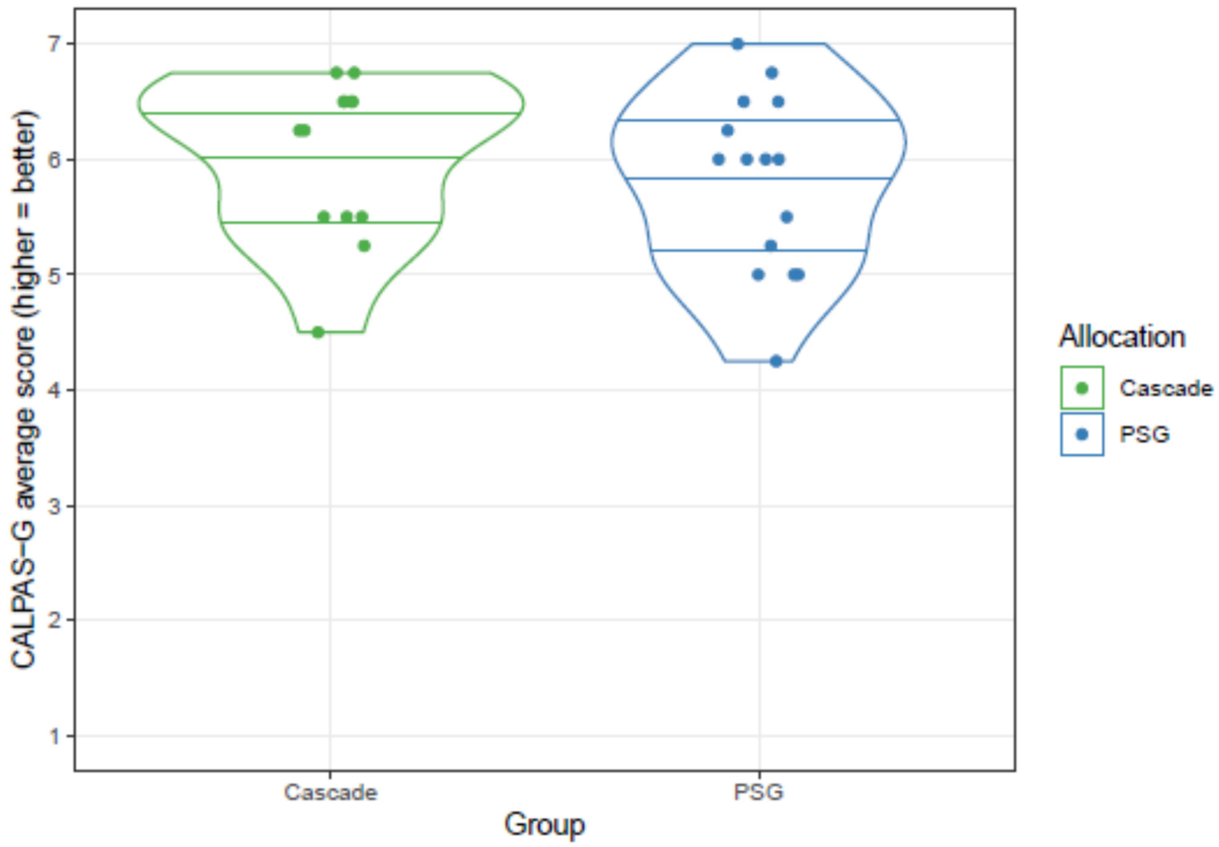

Supplementary Figure S2. Overall group cohesion ratings for both Cascade and the peer-support groups post-intervention

Note. The above violin plot shows the CALPAS-G scores for each study arm, with the shape width indicating a greater concentration of average CALPAS-G scores. PSG: Peer-support groups. Questions included: *'When important things came to mind, how often did you find yourself keeping them to yourself rather than sharing them with the group?'* (negatively scored), *'Do you feel accepted and respected by the group*

members for who you are?', *'How much did you find yourself thinking that the program was not the best way to get help with your concerns?'* (negatively scored), and *'How much did the group members help you gain a deeper understanding of your concerns?'*. Higher scores represented better group cohesion.

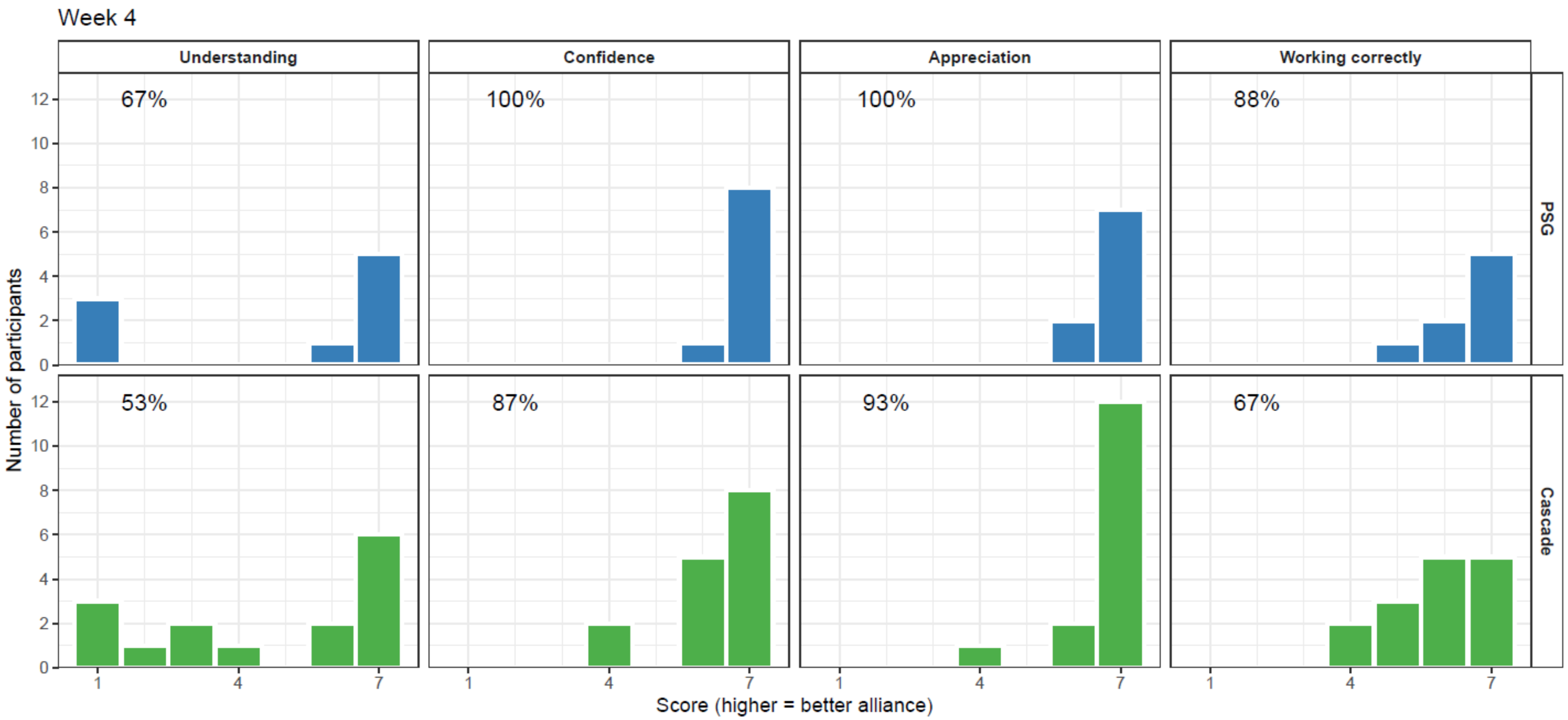

Supplementary Figure S3. Working Alliance Inventory ratings for both Cascade and the peer-support groups at week 4 (conclusion of the programs)

The above graph shows the distribution of scores for each Working Alliance Inventory domain in each arm (PSG: blue, Cascade: green) at week 4. The percentage shown in the top-left corner of each plot is the proportion of individuals who rated that domain as a 6 or 7: the best alliance scores. Participants answered four questions, rating the extent to which *'My group leader understands what I am trying to accomplish'*, *'I am confident of my group leader's ability to help me'*, *'I feel that my group leader appreciates me'*, and *'I believe that the way we are working with my concerns is correct'*. (1='doesn't correspond at all' to 7='corresponds exactly').

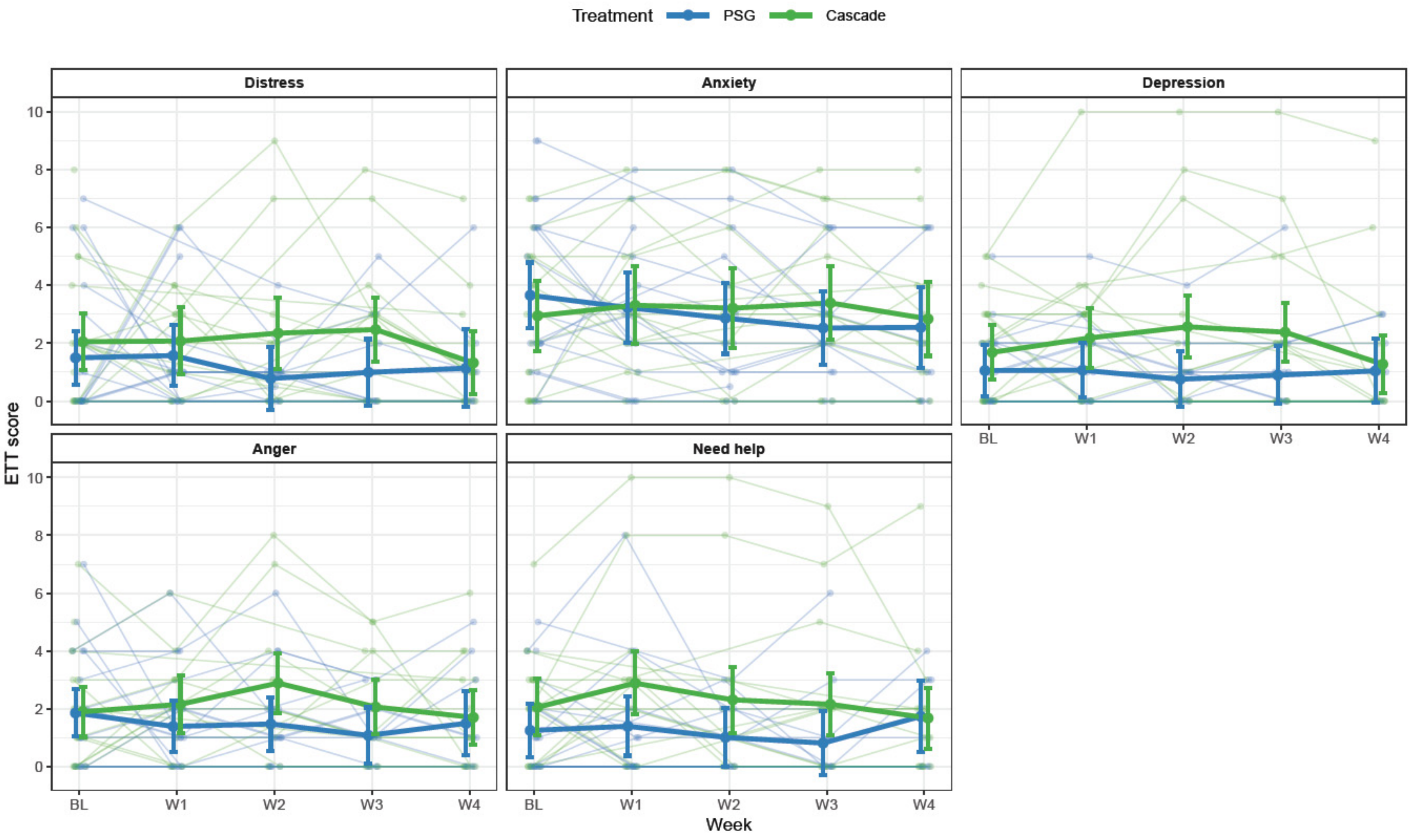

Treatment
PSG
Cascade
Distress
Anxiety
Depression
Anger
Need help
ETT score
Week
BL
W1
W2
W3
W4

Supplementary Figure S4. Emotion Thermometer ratings for Cascade and peer-support groups across each time-point

Note. *BL=baseline (Q1), W1=Week 1, following Session 1, W2=Week 2, following Session 2, W3=Week 3, following Session 3, W4=Week 4, following Session 4. ETT: Emotion Thermometer. PSG: Peer-support group.*
